# A new randomization procedure based on multiple covariates and applicable to parallel studies with simultaneous enrollment of all subjects prior to intervention

**DOI:** 10.1186/s12874-020-01085-w

**Published:** 2020-09-03

**Authors:** Eric D. Schoen, Suzan Wopereis

**Affiliations:** 1 TNO, Utrechtseweg 48, Zeist, 3700 AJ Netherlands; 2 Faculty of Bioengineering Sciences, KU Leuven, Kasteelpark Arenberg 30—Box 2456, Leuven, 3001 Belgium

**Keywords:** D-efficiency, Randomization, Blocking

## Abstract

**Background:**

Parallel intervention studies involving volunteers usually require a procedure to allocate the subjects to study-arms. Statistical models to evaluate the different outcomes of the study-arms will include study-arm as a factor along with any covariate that might affect the results. To ensure that the effects of the covariates are confounded to the least possible extent with the effects of the arms, stratified randomization can be applied. However, there is at present no clear-cut procedure when there are multiple covariates.

**Methods:**

For parallel study designs with simultaneous enrollment of all subjects prior to intervention, we propose a D-optimal blocking procedure to allocate subjects with known values of the covariates to the study arms. We prove that the procedure minimizes the variances of the baseline differences between the arms corrected for the covariates. The procedure uses standard statistical software.

**Results:**

We demonstrate the potential of the method by an application to a human parallel nutritional intervention trial with three arms and 162 healthy volunteers. The covariates were gender, age, body mass index, an initial composite health score, and a categorical indicator called first-visit group, defining groups of volunteers who visit the clinical centre on the same day (17 groups). Volunteers were allocated equally to the study-arms by the D-optimal blocking procedure. The D-efficiency of the model connecting an outcome with the study-arms and correcting for the covariates equals 99.2%. We simulated 10,000 random allocations of subjects to arms either unstratified or stratified by first-visit group. Intervals covering the middle 95% of the D-efficiencies for these allocations were [82.0, 92.0] and [93.2, 98.4], respectively.

**Conclusions:**

Allocation of volunteers to study-arms with a D-optimal blocking procedure with the values of the covariates as inputs substantially improves the efficiency of the statistical model that connects the response with the study arms and corrects for the covariates.

**Trial registration:**

Dutch Trial Register NL7054 (NTR7259). Registered May 15, 2018.

## Background

The motivating example for this paper is a clinical study to assess the health effects of generic versus personalized nutrition advice in healthy volunteers. There were three study arms: (1) a control arm where no advice was offered, (2) a generic nutrition advice arm and (3) a personalized nutrition advice arm. The study included a total of 162 subjects. Each of the subjects visited the clinical center three times. The initial visit involved collecting baseline information of the subjects such as anthropometrics and demographic data. Subjects furthermore consumed a mixed meal challenge drink and multiple blood samples were collected before and after consumption of the challenge drink to determine the physiological response. Based on this response, an initial health score was calculated [1]. A key feature of this study is the simultaneous availability of the baseline information for all 162 subjects prior to continuation with the intervention, which started at the second visit. The primary outcome of the study is the relation between the study arm and changes in health score between second and third visit.

Clinical studies such as the present one usually focus on comparing the effect of different interventions or treatments of the subjects. The main instrument for the comparison is a statistical model that links clinical outcomes to the intervention or treatment that was applied. An important concern when evaluating the treatment differences is that unadjusted treatment effects may be affected by random imbalances. For example, if subjects allocated to one of the arms are on average older than those allocated to another arm, the crude difference in average responses of these arms may be related to age rather than to the intervention.

There are well-known methods to correct the treatment effects for random imbalances of covariates. First, for subject-specific covariates, one could consider using a cross-over or within-subjects design, in which any subject receives all of the treatments, separated by a wash-out period. However, the treatments may be incompatible with such a design. For example, lifestyle interventions such as in the motivating example should involve a between-subjects rather than a within-subjects design, because effects are not easily washed out.

For parallel or between-subjects studies, each participant is allocated to one of the arms. Imbalance due to covariates can be reduced in two ways. First, the randomization procedure to allocate the subjects to the arms could be made to address specific sources of imbalance. For example, one could use a stratified randomization [2] to reduce imbalance due to age. This involves defining age classes, determining the age class for each subject and randomizing the allocation of the subjects of each age class to the arms of the study. As a consequence, the arms of the study have about the same average value of the covariate involved so that differences in treatment outcome means hardly need adjustment for differences in a covariate among the arms. Stratified randomization is a study design-based measure to reduce imbalance.

The second way to reduce imbalance in between-subjects studies is to include the potential sources of imbalance in the statistical model to evaluate study outcomes. The statistical model then provides estimates of the treatment effects corrected for covariates such as age. Inclusion of the covariate in the statistical model in addition to stratified randomization is hardly needed to remove imbalance, but it does reduce the unexplained variation within the arms. Therefore, the effects of the different treatments are estimated more precisely than without the inclusion of the covariate in the model, so that the power to detect intervention effects is increased.

Provided that there is a simultaneous enrollment of all subjects prior to starting the interventions, it might be possible to define homogeneous study groups based on multiple covariates. When these groups are sufficiently large, stratified randomization could proceed in the same way as for a single covariate. However, with an increasing number of covariates the groups become too small for a stratified randomization. Nevertheless, it is desirable that the arms of a human study have about the same average value for all of the covariates, or, in case of a categorical covariate, shows a proportional distribution of that covariate for the respective arms. The motivating example includes gender, age, body mass index (BMI), and a composite health score as continuous covariates. Subsequently, a categorical covariate called first-visit group defines groups of subjects whose initial visit of the clinical center was on the same day. There are 17 different first-visit groups so that a stratified randomization addressing all the covariates would be impossible to perform.

The purpose of this paper is to propose a method to allocate subjects to the arms of a study using multiple covariates that are simultaneously available for all subjects before intervention starts in any subject. The method is model-based such that the variances of the intervention effects corrected for the covariate are minimized. It can be conducted using commercially available statistical software, requiring as input a list specifying for each subject the covariates that need to be addressed, and the number of arms in the study. The output is an allocation table where each subject is allocated to one of the arms. We further propose a quantitative measure of the effectiveness of any allocation. We show the effectiveness of the proposed allocation method for the motivating example using both this measure and tables of mean values or frequencies of the covariates for the three study-arms. We compare the effectiveness of our procedure with results on 10,000 completely random allocations and 10,000 stratified random allocations within each of the 17 levels of the first-visit group. Finally, we sum up benefits and possible drawbacks of the procedure.

## Methods

### Allocation procedure

Our method to allocate subjects to the arms of a study is based on a statistical model that relates any parameter of interest to the arms of the study and the covariates considered to be relevant for that study. We assume that these parameters are continuous so that we can use linear-model methodology. Let *y* be the *N*×1 vector of observed values of these parameters. Further, let *X* be the *N*×*p* matrix of covariates and *T* the *N*×(*t*−1) matrix of contrasts modelling the differences between the *t* arms of the study. The statistical model of the data is then

1 $$ y=X\beta+T \gamma+\epsilon,  $$

where *β* is a *p*×1 vector of coefficients corresponding to the covariates that includes the intercept, *γ* is a (*t*−1)×1 vector of coefficients quantifying the differences between the arms, *ε*∼*N*(0,*σ*^2^*I*_*N*_) is a vector of identically and independently distributed normal variables with mean 0 and variance *σ*^2^, and *I*_*N*_ is the *N*×*N* identity matrix.

The purpose of the study is to estimate *γ* as precisely as possible. Atkinson, Donev and Tobias [3] show that the precision of the estimator of *γ* is maximized if

2 $$ \mathcal{D}_{s}=|T^{T} (I-X(X^{T} X)^{-1} X^{T})T|  $$

is maximized, where |.| denotes the determinant. This is the determinant of the residual sums of squares and products matrix after regressing the *t*−1 contrast vectors in *T* that make up the differences between the arms of the study on the covariates collected in *X*. Denoting the full matrix of parameters with *F*=[*XT*], it can be shown that (2) is equivalent to

3 $$ \mathcal{D}_{s}=|F^{T} F|/|X^{T} X|  $$

The matrix *X* is fixed at the time of allocation of the subjects to the arms of the study. The only way to affect $\mathcal {D}_{s}$ is in the allocation of the subjects to the arms as expressed with the matrix *T*. We propose optimizing this allocation using the blocking procedure of Cook and Nachtsheim [4].

The blocking procedure [4] groups the rows of a pre-existing matrix *X* with contrast columns of primary interest into groups, or blocks, of a specified size such that the precision of the estimator of the effects of primary interest is maximized. Denoting the blocking contrast matrix by *B* and using *G*=[*BX*], the procedure maximizes

4 $$ D^{*}=|G^{T}G|/|B^{T}B|.  $$

The blocking factor is a categorical covariate added by the procedure. Note that |*B*^*T*^*B*| is fixed by the block sizes. For the allocation of subjects to arms, however, we have a pre-existing matrix of covariates *X* that are not of primary interest, and we want to allocate the rows to groups corresponding to the arms. For this purpose, we also use the matrix *B* returned by the blocking procedure, replace the symbol *B* by *T* and the symbol *G* by *F*. Equation (4) is thus re-expressed as *D*^∗^=|*F*^*T*^*F*|/|*T*^*T*^*T*|. If we fix the number of subjects in each arm, we fix |*T*^*T*^*T*|. Therefore, |*F*^*T*^*F*| is maximized by the procedure. By (3), maximization of |*F*^*T*^*F*| implies maximization of $\mathcal {D}_{s}$ so that the procedure can be used to optimize the allocation of subjects to the arms of a study as well, provided that the numbers of subjects in each of the arms are predetermined.

One of the favorable properties of unrestricted randomization is that the distributions for both observed and unobserved covariates in the respective treatment groups are statistically comparable. Our proposed blocking procedure directly deals with comparability of observed covariates included in the matrix *X* and indirectly with unobserved covariates that are correlated with the observed covariates in *X*. For other covariates, the procedure retains a random element, because the $\mathcal {D}_{s}$ optimal allocation starts with a random allocation of the treatments to the rows of the matrix of covariates. Indeed, independent applications of the procedure result in different allocations that are, however, equally efficient (see next section for a measure of effectiveness).

### Measuring effectiveness

By writing the determinant in (2) as

5 $$ \mathcal{D}_{s}=|T^{T}T - T^{T}X(X^{T} X)^{-1} X^{T} T|,  $$

it can be seen that the maximum is reached if *X* and *T* are orthogonal to each other, and *D*_*s*_=|*T*^*T*^|. Without loss of generality, *T* can be made to include *t*−1 orthogonal contrasts with length $\sqrt {N}$. Therefore, |*T*^*T*^*T*|=*N*^*t*−1^. Following Schoen [5], we define the *D*_*s*_ efficiency of the study design with respect to the covariates as

6 $$ D_{s}=\mathcal{D}_{s}^{1/(t-1)}/N.  $$

Its value equals 1 if the allocation is such that the treatment contrasts are orthogonal to the covariates. Its value equals 0 if one or both of the treatment contrasts are completely aliased with one of the covariates.

The *D*_*s*_ criterion clearly depends on *X*^*T*^*X* and *X*^*T*^*T*,*T*^*T*^*T* being constant. It cannot reflect *X*^*T*^*Y*, because it works on existing data, while *Y* belongs to future data, collected after the allocation of the subjects to the arms of the study. Correlation between the covariates in *X* and the response *Y* is taken into account in the statistical model for the collected data.

## Results

### Performance of the allocation procedure

The motivating example detailed at the start of this paper involves three study arms and a total of 162 subjects. Each of the subjects visited the clinical center three times. At the initial visit, baseline information of the subjects was collected such as BMI, age and gender. Subjects furthermore consumed a mixed meal challenge drink and multiple blood samples were obtained before and afterwards to analyse the physiological response. Based on this response, an initial health score was calculated [1]. A key feature of this study is the simultaneous availability of the baseline information for all 162 subjects prior to the start of the intervention, so that this information can be employed in the randomization. The intervention started on the second visit to the clinical center. The main interest of the study is in the relation between the study arm and changes in health score between second and third visit.

Gender, age, BMI and initial health score may affect the change in health parameters between the second and third visit. Therefore, they should be included as covariates in a statistical model relating the change in health score between second and third visit to the arm of the study. We decided also to include the grouping variable that indicates which participants initially visited the clinical center on the same day. There are 17 first-visit groups indicated by this variable. These groups of subjects remain largely the same for the second and third visits. Therefore, including the first-visit group as a variable in a statistical model accounts for different changes in health parameters for people starting early in the study when compared to those starting later on, for example by seasonal variation.

In view of the main interest of the study, the differences between the arms as regards the changes in health parameters between the second and third visit should be confounded to the least possible extent with differences between gender, age, BMI, initial health score and first-visit group. The matrix *X* in Eq. 1 thus includes one column for each of the parameters gender, age, BMI and initial health score and 17 columns indicating whether a subject belongs to first-visit group 1 up to 17 (an entry 1 for subject *i* in column *j* indicates that subject *i* belongs to first-visit group *j*).The matrix *T* is a 162×2 matrix of normalized contrasts making up the differences between the 3 arms of the study. An allocation of subjects to the arms of the study corresponds to a permutation in the rows of *T* against a fixed matrix *X*. We impose the restriction that each arm is to include 54 subjects. Therefore, |*T*^*T*^*T*| in Eq. (4) equals 162^2^.

We used SAS/QC procedure OPTEX with 5000 iterations to allocate the subjects to the arms. The *D*_*s*_-efficiency of the allocation is 0.992. Table 1 shows the distribution over the arms for female (F) and male (M) subjects. The table shows that the 104 female subjects are allocated as evenly as possible to the three arms. The same is the case for the 58 male subjects. Neither 104 nor 58 is divisible by 3 so that the distribution cannot be perfectly even. The distribution is such that each arm includes 54 subjects and the ratio of female over male is as close to constant as possible. Table 2 shows the distribution over the arms for each first-visit group. Note that these groups are of unequal size. It is clear that the subjects of one and the same first-visit group are allocated as evenly as possible to the three arms. Finally, the means and standard deviations of the continuous covariates age, BMI and initial health score are shown in Table 3. The three arms have nearly the same mean values of these covariates, while their standard deviations are also close. We conclude that the allocation procedure was very successful in returning an efficient study design.

**Table 1:** Allocation of subjects to study arms according to gender in the motivating example

Gender	Arm		
	A	B	C
F	34	35	35
M	20	19	19

The table shows the numbers of female (F) and male (M) subjects allocated to the arms A, B and C, respectively

**Table 2:** Allocation of subjects to study-arms according to first-visit group in the motivating example

Visit	Arm		
	A	B	C
1	3	4	3
2	3	3	4
3	4	4	3
4	3	2	2
5	4	3	4
6	3	3	4
7	3	2	2
8	4	4	3
9	2	3	3
10	3	3	3
11	3	3	3
12	3	4	4
13	3	3	4
14	4	3	3
15	4	4	4
16	2	2	2
17	3	4	3

A first-visit group comprises the subjects that had their intake visit on the same day. The table shows for each first-visit group the numbers of subjects allocated to the arms A, B and C, respectively

**Table 3:** Allocation of subjects to study-arms according to age, BMI and initial health score in the motivating example

Covariate	Arm					
	A		B		C	
	mean	sd	mean	sd	mean	sd
Age	42.63	12.59	42.33	12.36	42.93	11.87
BMI	25.28	3.15	25.31	3.68	25.27	3.97
Health Score	1.476	0.135	1.475	0.121	1.478	0.133

The table shows means and standard deviations of the covariates in the arms A, B and C, respectively

Finally, we checked that repeated applications of the procedure, using different random seeds, indeed result in different allocations with the same $\mathcal {D}_{s}$ efficiency. We conclude that the allocation procedure retains features of stratified randomization.

## Discussion

### Comparison with complete and restricted randomization

To assess the amount of improvement due to the proposed procedure, we performed 10,000 random allocations of the subjects to arms and evaluated the *D*_*s*_ efficiency (6) of each of the resulting 10,000 study designs with respect to the covariates in that design. We considered a completely random allocation and a random allocation stratified by first-visit group. For the latter case, we had to assume that, for each first-visit group, the numbers of subjects assigned to each of the arms correspond to the numbers in Table 2.

**Fig. 1:**
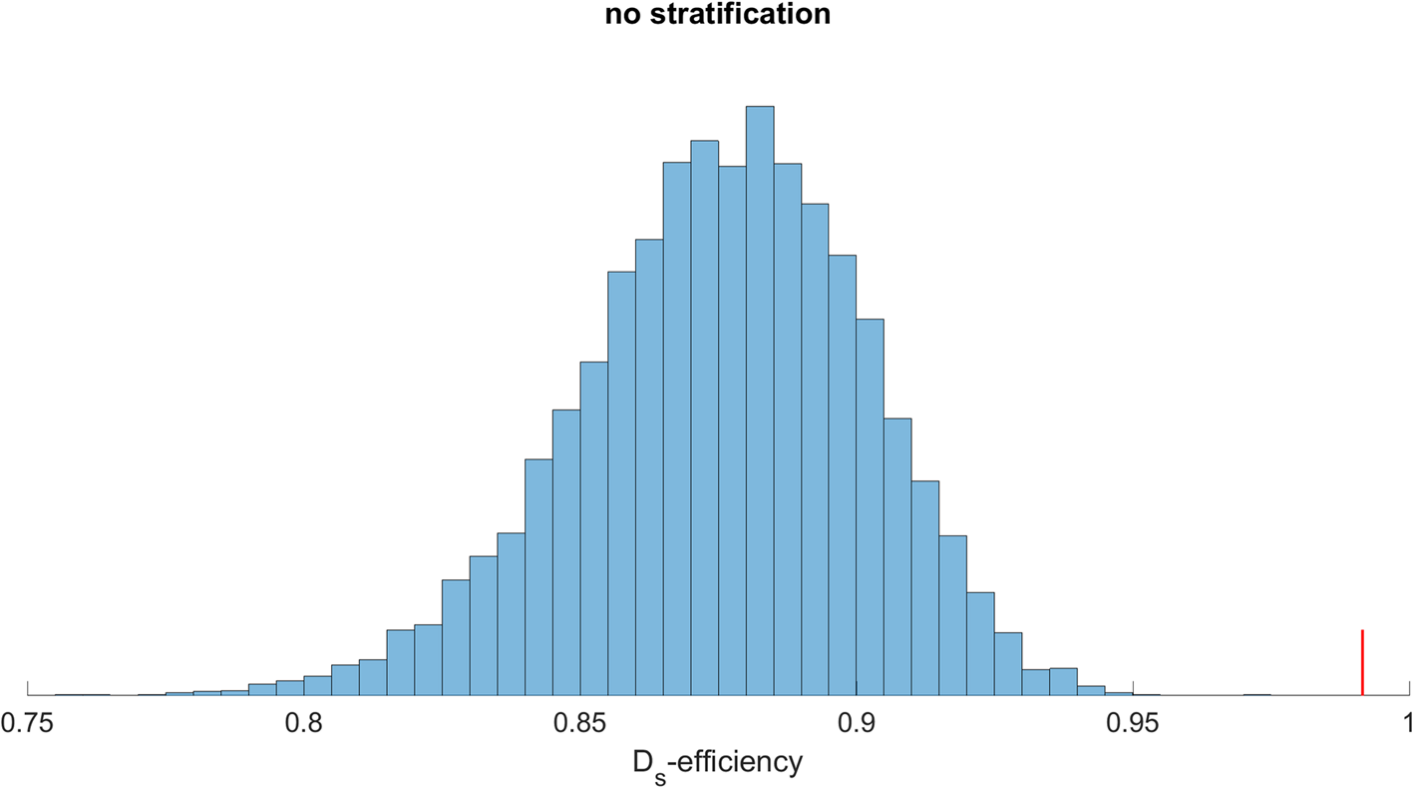
D _*s*_-efficiencies for potential complete randomizations in the motivating example. Ten thousand study designs are generated by random allocation of subject to arms, not stratified by any covariate. The figure shows *D*_*s*_ efficiencies of the 10,000 study designs with respect to covariates gender, age, BMI, a composite health score and membership of one of 17 first-visit groups as covariates. The red bar denotes the efficiency obtained with the proposed procedure, which addresses all covariates simultaneously

**Fig. 2:**
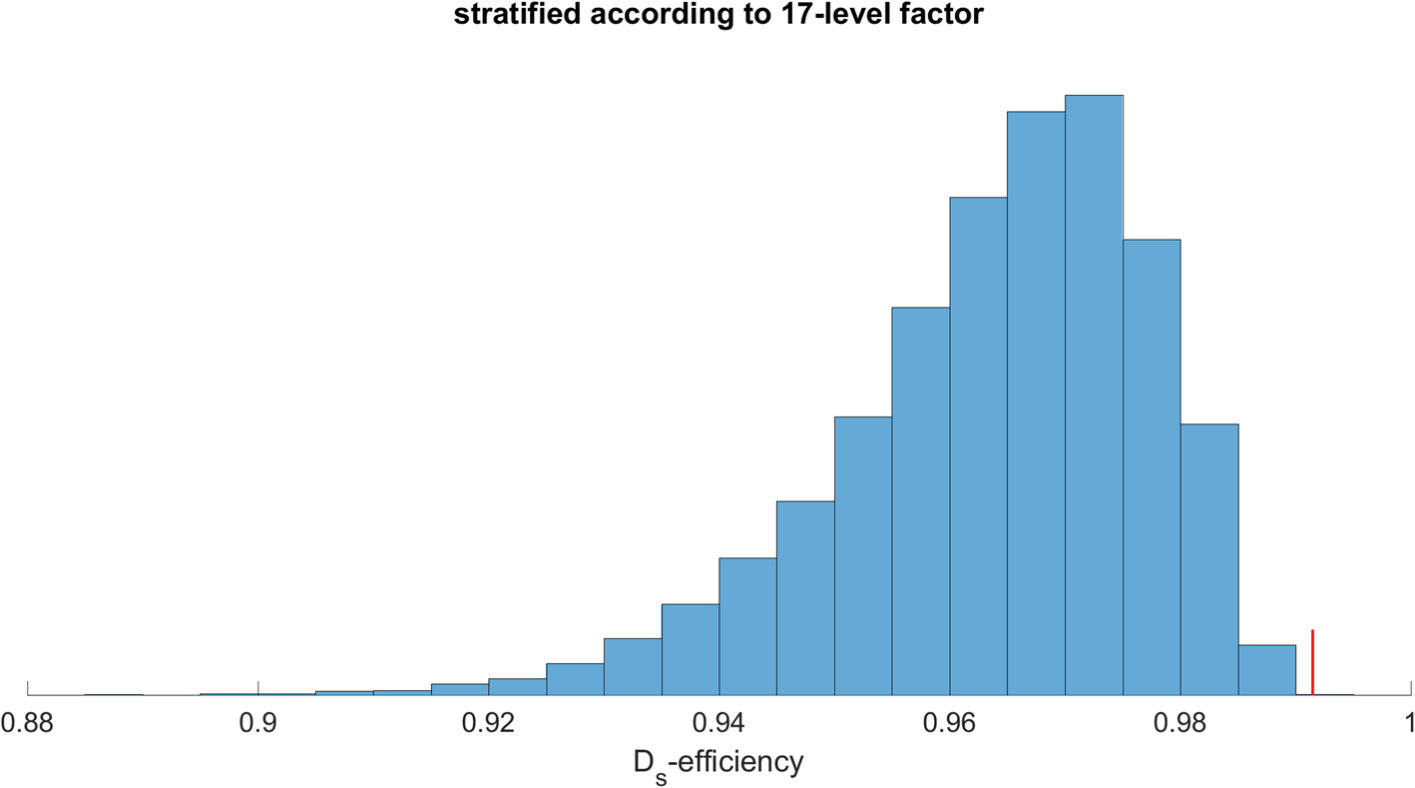
D _*s*_-efficiencies for potential stratified randomizations in the motivating example. Ten thousand study designs are generated by random allocation of subject to arms, stratified by first-visit group. The figure shows *D*_*s*_ efficiencies of the 10,000 study designs with respect to covariates gender, age, BMI, a composite health score and membership of one of 17 first-visit groups as covariates. The red bar denotes the efficiency obtained with the proposed procedure, which addresses all covariates simultaneously

The results of the completely random and stratified random allocations are shown in Figs. 1 and 2, respectively. In both figures, the blue bars show a frequency distribution of 10,000 allocations. The proposed procedure addresses all covariates simultaneously. The small red bar denotes the *D*_*s*_-efficiency of 99.2 obtained in this way. Intervals covering 95% of the *D*_*s*_-efficiencies for the completely random and stratified random allocations were [0.80, 0.90] and [0.93, 0.98], respectively. Maximum values were 0.97 and 0.99 respectively. We conclude that it is very likely that a random allocation returns a substantially worse *D*_*s*_-efficiency for the study design than a model-based allocation. Even in case the numbers of subjects per arm are predetermined for each first-visit group, the stratified randomization generally produces inferior study designs.

In principle, allocation of volunteers to study-arms could also be achieved by methods that are specifically designed to balance out sequential experiments. These methods might be applied to studies where all the covariates are known prior to actual treatment by mimicking sequential entrance in the study and allocating the volunteers one by one. We briefly discuss two examples. First, Sajobi et al. [6] provide a heuristic method to balance multiple covariates as much as is feasible over the arms of the study. Their method employs an updated table of the current distribution of each individual covariate over the arms to allocate a new subject to one of these arms. Instead, our method operates directly on the precision of the parameter estimators modeling the differences between the arms when corrected for the joint effects of the covariates.

As a second example, we mention a biased coin type of randomization [7], which involves counts *x*_*ijk*_ quantifying the number of patients with level *j* of prognostic factor *i* who have been assigned to treatment *k*. Operationalizing the procedure requires a measure of the amount of variation of the counts for each prognostic factor within each of the treatments, as well as combining the amounts of variation of the respective prognostic factors to one figure per treatment. In addition, based on the combined measure, allocation probabilities for a new subject to each of the treatments have to be defined. All choices required seem to some extent arbitrary. In contrast, the proposed method has a clearly defined objective function.

Finally, one of the referees of this paper suggested that, if historical estimates of *β* in Eq. (1) are available, then stratified assignment could be based on quartiles, or possibly deciles, of *Xβ*. This would be highly beneficial, since using these quantiles permits a stratified randomization that directly addresses the expected differences in response levels due to the covariates. However, for covariates that bear on the experimental conduct, such as the first-visit group in the motivating example, there are no historical estimates. In that case, we can apply the proposed procedure on the categorical covariate defined by the quantiles of *Xβ* and the covariates with no available historical estimates of their effects.

### Restriction to specific study designs

The allocation procedure proposed here is appropriate for a parallel study design in which the subjects are simultaneously allocated to the treatment arms. This implies that the procedure is not compatible with studies where an immediate choice among treatments is required such as sequential clinical trials that enrol subjects gradually.

The procedure is also not compatible with a cross-over design in which each subject receives all the treatments sequentially. A correction for the covariates determined prior to the actual start of the study would affect these treatments in the same amount. As a result, treatment differences in these cross-over studies are unaffected by covariates measured at the start. If the subjects do not receive all of the treatments, most of the analyses will still be based on within-subject differences which are not affected by the covariates of a subject as measured at the start of the study. Our procedure might be helpful, though, if one requires to use inter-subject information for the treatment differences.

### Multiple endpoints

Our procedure is designed for studies with a single primary endpoint. When there are two or more primary endpoints, there may be specific covariates for each of the endpoints. A given allocation of subjects to study-arms would then result in *D*_*s*_ efficiencies (6) for each of the endpoints. An interesting subject for further research is the adaptation of our procedure for the multiple-endpoint case.

## Conclusions

In this paper, we proposed a model-based procedure to allocate subjects with known values of a set of covariates to the study-arms of a parallel study with simultaneous enrollment of all subjects prior to intervention. The procedure can be viewed as an extension of stratified randomization to the case of multiple covariates. It operates using standard statistical software. Its goal function is such that the allocation results in a maximum precision for the effects of the study-arms after correction for the covariates. The success of the procedure can be measured by the *D*_*s*_-efficiency of the resulting design and by summary tables classified by the covariate values and the study-arm. An application to a human intervention trial with three arms and 162 volunteers showed that the procedure is highly effective.

## Supplementary information

**Additional file 1** SAS code to allocate the subjects to the three arms of the motivating example study.

## Data Availability

Data used for the allocation are available upon reasonable request. SAS code for the allocation is provided as a supplementary file.

## Abbreviations

BMI: Body mass index
